# Draft genome sequence of a methicillin-resistant *Staphylococcus aureus* MRSA_GSTU_PS9_BD strain isolated from a patient’s wound in Bangladesh

**DOI:** 10.1128/mra.00608-26

**Published:** 2026-06-29

**Authors:** Abdul Malek, Sazzad Hossain Sagor, Anika Tasnim, Md. Naim, Nizam Uddin, Rabeya Khanam, Md. Yamun Hasan, Ismatara Munia, Md. Abdurrahim, Md. Sahabuddin

**Affiliations:** 1 Department of Biotechnology and Genetic Engineering, Faculty of Life Science, Gopalganj Science and Technology University 421965, Gopalganj, Bangladesh; 2 Biochemistry and Molecular Biology Research Division, Biomedical and Toxicological Research Institute (BTRI), Bangladesh Council of Scientific and Industrial Research 130051, Dhaka, Bangladesh; 3 Genomic Research Laboratory, Bangladesh Council of Scientific and Industrial Research 130051, Dhaka, Bangladesh; 4 Biotechnology and Genetic Engineering Discipline, Life Science School, Khulna University 247289, Khulna, Bangladesh; Rochester Institute of Technology, Rochester, New York, USA

**Keywords:** antibiotic resistance, *mecA*, *Staphylococcus aureus*, methicillin resistance, genomes

## Abstract

We report the draft genome sequence of the methicillin-resistant *Staphylococcus aureus* (MRSA) strain MRSA_GSTU_PS9_BD, isolated from a patient’s wound infection in Bangladesh. The genome harbors 123 and 22 predicted virulence factors and antimicrobial resistance genes, respectively, with a 91.86% probability of being a human pathogen.

## ANNOUNCEMENT

Methicillin-resistant *Staphylococcus aureus* (MRSA) is a bacterium that poses a serious threat in healthcare facilities and community settings due to its resistance to multiple antibiotics. It is a global concern that requires careful consideration and effective therapeutic approaches (1, 2). MRSA strains are currently causing a significant 25%–50% of clinical *S. aureus* infections in different countries (3). Annually, there are over 80,000 new cases of MRSA acquired in hospitals, along with more than 11,000 deaths related to hospital-associated MRSA (4).

*Staphylococcus aureus* MRSA_GSTU_PS9_BD was isolated from wound infections in Gopalganj Sadar Hospital, Bangladesh in 2024. Wound swabs were collected aseptically in Falcon tubes containing phosphate-buffered saline and transferred to the Microbiology Laboratory at the Department of Biotechnology and Genetic Engineering, Gopalganj Science and Technology University, Gopalganj-8105, Bangladesh, where presumptive identification of *S. aureus* was first performed by standard cultural, morphological, biochemical, and molecular assays. Antimicrobial susceptibility testing was performed via the Kirby-Bauer disk diffusion technique. Methicillin resistance was confirmed by PCR targeting the *mecA* gene. Among the identified isolates, *Staphylococcus aureus* MRSA_GSTU_PS9_BD exhibited extensive multidrug resistance and was therefore selected for whole-genome sequencing (WGS) to evaluate its antimicrobial resistance patterns, virulence profiles, and genomic characteristics essential to public health surveillance. *S. aureus* strain MRSA_GSTU_PS9_BD was cultured overnight on nutrient agar at 37°C. The isolate was submitted for WGS to the Genome Center of the International Center for Diarrhoeal Disease Research, Bangladesh. Genomic DNA was extracted using the Wizard Genomic DNA Purification Kit (cat. A1620; Promega, Madison, WI, USA), and the DNA library was prepared using Illumina DNA Prep (Illumina, San Diego, CA, USA). Sequencing was carried out on the Illumina NextSeq 2000 platform, generating Illumina paired-end raw 2 × 150 bp reads. The raw reads were quality controlled using FastQC v0.12.1 (5) and trimmed using fastp v0.23.4 (6). *De novo* assembly was performed using SPAdes v3.15.5 (7, 8) and classified using Kraken2 v2.1.3 (9).

The detailed genomic characteristics of the isolated *S. aureus* strain MRSA_GSTU_PS9_BD are summarized in Table 1. The BV-BRC (10) and the NCBI Prokaryotic Genome Annotation Pipeline v6.10 (11) were used to resolve a final genome of 34 contigs with a total length of 2,846,816 bp and a GC content of 32.68%. Analysis of the assembled contig with PathogenFinder v2.0 (12) showed the isolate had a 91.86% probability of being a human pathogen. Analysis with RGI main from CARD v4.0.2 (13) identified 22 AMR genes, including *blaZ*, *mecA*, *erm(C)*, *tet(K)*, and *dfrG*. Virulence Factors Database (14) predicted 123 VFs genes, including *coa*, *hlgA-C*, *lukF-PV*, *lukS-PV*, and *sep*. The MLSTFinder v1.0.1 (15) from the CGE web server revealed that our sequence type is ST2884, which was the most prevalent lineage of *S. aureus* in this region. Default parameters were applied for all software unless otherwise specified.

**TABLE 1:** Genomic characteristics of *S. aureus* strain MRSA_GSTU_PS9_BD isolated from wound infections

Attributes	Values
BioProject accession no.	PRJNA1445818
BioSample accession no.	SAMN56799133
GeneBank accession no.	JBWQPW000000000
SRA accession no.	SRR37920994
Contigs	34
Genome size (bp)	2,846,816
GC content (%)	32.68
Contig L50	3
Contig N50	284,088
Coverage (×)	125.6
Sequence completeness (%)	99.51
Genes (total)	2,895
CDS (total)	2,832
Genes (coding)	2,750
Total RNAs	63
No. of tRNAs	56
No. of rRNAs	3
No. of ncRNAs	4
No. of pseudogenes (total)	82
Pseudogenes (ambiguous residues)	0
Pseudogenes (frameshifted)	35
Pseudogenes (incomplete)	43
Pseudogenes (internal stop)	19
No.of hypothetical proteins	561
No. of AMR genes	22
No. of VFs genes	123
No. of intact prophage regions	3

## Data Availability

The Whole-Genome Shotgun Sequencing Project for the *Staphylococcus aureus* strain MRSA_GSTU_PS9_BD has been deposited in DDBJ/ENA/GenBank under accession number JBWQPW000000000 and in the Sequence Read Archive under BioProject accession number PRJNA1445818.
